# Retraction: NeuroSuites: an online platform for running neuroscience, statistical, and machine learning tools

**DOI:** 10.3389/fninf.2024.1376953

**Published:** 2024-02-05

**Authors:** Frontiers Editorial Office

The journal retracts the 17th February 2023 article above.

In light of complaints received following publication of the original research article, Frontiers carried out a detailed investigation of the academic, ethical, and legal aspects of the work. This investigation did not identify any issues with the academic and ethical aspects of the study. It did, however, determine that the legal context is insufficiently clear and cannot be addressed through correction; the article is therefore retracted.

This retraction was approved by the Chief Executive Editor of Frontiers. The authors understand this decision, while they stand by their article and regret the limitations on academic freedom which can be caused by legal factors.

